# Autoinflammatory/Autoimmunity Syndrome Induced By Adjuvants (ASIA) Due to Silicone Incompatibility Syndrome

**DOI:** 10.1155/2021/5595739

**Published:** 2021-08-16

**Authors:** Genessis Maldonado, Roberto Guerrero, Maria Intriago, Carlos Rios

**Affiliations:** ^1^Loyola MacNeal Hospital, Internal Medicine Residency Program, Maywood, IL, USA; ^2^Universidad Espiritu Santo, Rheumatology Department, Guayaquil, Ecuador

## Abstract

The adjuvant-induced autoimmune syndrome (ASIA) is associated with a dysregulation of the innate and adaptive immune system after exposure to chemical compounds, including liquid paraffin, silicone gel, acrylamides, and hyaluronic acid. Due the increase of the use of these compounds in cosmetic procedures, the prevalence of this syndrome is increasing. We present the first report in Ecuador associated to ASIA after an elective silicone breast prosthesis procedure, manifested as polyarthralgia, positive antinuclear antibody, anticentromere antibody, and a moderate positive Sclero-70.

## 1. Introduction

A real-world analysis performed by Watad et al. showed an association between silicone breast implants (SBIs) and autoimmune/rheumatic disorders in a large population-based database, and the strongest association with SBI was evidenced for Sjogren's syndrome, systemic sclerosis, and sarcodoisis [1]. Adjuvant-induced autoimmune syndrome or ASIA was recognized in 2011 with the aim of studying the growing appearance of disorders characterized by an innate and adaptive dysregulation of the immune system after exposure to adjuvants [2]. These compounds include liquid paraffin, silicone gel, acrylamides, hyaluronic acid, aluminum, and methacrylate derivatives. Symptoms of ASIA due to silicone incompatibility syndrome are fatigue, arthralgias, and cognitive impairment/memory loss [3].

Due to the growing trend of cosmetic procedures, it is important to recognize this association. A systematic search of the possible cases reported in the country was carried out. So far, there are no reports of the ASIA syndrome. It would seem that this would be one of the first reports made in the country. In addition, there are no official national registries for ASIA syndrome triggered by silicone prostheses.

## 2. Case Report

A 32-year-old female patient presented a 5-month history of polyarthralgia. The patient was evaluated by the trauma department due to a meniscal injury, and she was referred to the rheumatology department due to the presence of positive antinuclear antibodies. On physical examination, she presented polyarthralgia with a predominance of metacarpophalanges without swelling. As a relevant antecedent, the patient refers she underwent an abdominoplasty and breast augmentation with silicone implants placement approximately 5 years ago. Information regarding the implant brand is unknown. Initial laboratory tests showed a 1 : 640 positive antinuclear antibody, anticentromere antibody and a moderate positive Sclero-70. Immunoglobulin and IgG subclasses were not available (Table 1).

## 3. Differential Diagnosis

Because it is a recently described condition, this must be a diagnosis of exclusion. Diagnostic criteria for this condition were developed by Shoenfeld et al. [2]. To make the established diagnosis, the patient must meet at least two of the major criteria or one major and two minor criteria (Table 2).

**Figure 1:**
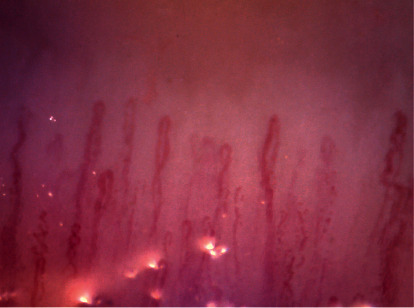
Normal pattern capillaroscopy adequate density without the presence of giant capillaries, avascular areas, or microhemorrhages.

The patient was found to have two major criteria and one minor criterion, confirming the presence of the ASIA syndrome. Regarding collagen disease studies, a video capillaroscopy was performed, in which a pattern of normal characteristics was observed (Figure 1).

It was recommended to remove the breast implants, and the patient underwent removal of prosthesis approximately 6 months later; however, the presence of polyarthralgias and Raynaud's phenomenon were continued to be monitored.

## 4. Discussion

Silicone implants have been used since the 60s as medical devices, whether they are intraocular lenses, heart valves, testicular, and joint and/or breast prostheses. Although their safety has been proven by several studies and public health institutions over the years, the presence of ASIA syndrome is possible in genetically prone patients.

The association that exists between breast implants and the immune system lies in the production and activation of B and T cells and increased IgG antibodies and Th1/Th17 cells in the silicone capsule [4]. Furthermore, this material can induce an immunogenic response through cross reactions with natural glycosaminoglycans and molecules that contain silicone in connective tissue [4].

Because of this, the international registry of ASIA syndrome was created [5], and at the moment, there have been approximately 500 registered cases studied [6]; the majority were women, of which 69% developed defined autoimmune diseases (mixed connective tissue disease, Sjögren's syndrome, and scleroderma among others). 12.5% were exposed to silicone implants. The two most common diseases reported after exposure were differentiated connective tissue disease and Sjogren's syndrome [6]. Furthermore, the presence of positive antibodies was evidenced in 54.4% of the cases.

The set of symptoms that develop after exposure to silicone implants is known as siliconosis, and according to the reports by Boer et al., symptoms improve after implants are removed [7]; however, data from the work of Colaris et al. showed that the removal of the prosthesis temporarily alleviated the symptoms [3], and the authors also described that patients with ASIA and vitamin D deficiency were more prone to develop antibodies, as the patient presented in this case [3]. Wronski et al. reported a case of a 48-year-old woman with a history of breast implants and postexposure ASIA symptoms, with the presence of antinuclear antibodies, anticitrullinate, and Raynaud's phenomenon, and the patient underwent excision of the implants and the symptoms improved. However, Raynaud's phenomenon continued to be present, and the patient developed Hashimoto's thyroiditis [8]. Mejis et al. described improvement of systemic sclerosis symptoms after removal of the SBIs. Furthermore, the management of our patient will depend on the evolution after removal of the prostheses. Due to the presence of antinuclear, anticentromere, and Sclero-70 antibodies, it is important to evaluate the possible development of systemic sclerosis, hence the importance of knowing the relationship between silicone implants and the development of autoimmune diseases.

## 5. Conclusions

This is the first case of ASIA due to silicone incompatibility syndrome described in Ecuador. The appearance of symptoms such as myalgias, fatigue, morning stiffness, and constitutional symptoms after exposure to breast implants should be evaluated in order to rule out ASIA syndrome. Those patients with a ruptured breast implant tend to have a more aggressive presentation compared to those with implants without alterations. Proper management is the removal of the implants, and if symptoms persist, disease-modifying drugs, hydroxychloroquine, and glucocorticoids can be used.

## Figures and Tables

**Table 1:** Laboratory values.

Laboratory studies	Results	Reference values
Inflammatory markers		
C-reactive protein	10.73 mg/dL	0.00–5.00 mg/dl
Immunological		
Antinuclear antibodies (ANA) HEp-2-IF	Positive 1: 640, centromeric pattern to immunofluorescence	Positive ≥ 1 : 40
Anti-DNA ds, IF	Negative	Positive ≥ 1 : 10
Antimicrosomal antibodies (TPO)	0.44 IU/ml	0.00–4.61
Interleukin-6 (IL-6)	4.30 pg/ml	0–6.50 pg/ml
Sclero-70 antibodies	46.67 U	<20: negative 20–39: positive-weak 40–80: positive-moderate >80: positive-strong
Anti-SSB (LA) (ELISA)	1.86 U	<16: negative 16–20: equivocal >20: positive
Anti-SM (ELISA)	7.06 U	<20: negative 20–39: positive-weak 40–80: positive-moderate >80: positive-strong
Anti-RNP (ELISA)	5.00 U	<20: negative 20–39: positive-weak 40–80: positive-moderate >80: positive-strong
Anti-JO	3.60 U	<20: negative 20–39: positive-weak 40–80: positive-moderate >80: positive-strong
Rheumatoid factor- nephelometry	<20.0 IU/mg	0.00–20.0 IU/mg
Citrullinated cyclic peptide antibody	0.60 U/mL	Positive: <5.0 U/mL
Total vitamin D	25.80 ng/ml	≥30 ng/dl
Antithyroglobulin antibodies	5.48 IU/ml	<4.5 UI/mL
Complement C3 nephelometry	115.00 mg/dL	79.00–152.00 mg/dL
Complement C4 nephelometry	21.60 mg/dL	16.00–38.00 mg/dL
Hormonal Profile		
Intact parathyroid hormone	80 pg/mL	18–116 pg/mL
TSH- thyroid stimulating hormone	0.7273 uUI/mL	Adults: 0.35–4.95
T4- total thyroxine	7.57 *μ*g/dL	Adults: 4.90–11.70
T3- total triiodothyronine	1.19 nmol/L	Adults: 0.89–2.44
Virology		
Hepatitis delta IgM (EIA)	Negative	
HBsAg- hepatitis B surface antigen	Not reactive	
HBcAc- hepatitis B, anticore IgG/IgM	Not reactive	
Hepatitis C, antibodies	0.03	Reactive > 1

**Table 2:** Diagnostic criteria for ASIA syndrome.

Major criteria	Minor criteria
Exposure to an external stimulus (adjuvants) before clinical manifestations	The appearance of autoantibodies or antibodies directed to the suspected adjuvant

Appearance of “typical” clinical manifestations (i) Myalgia, myositis, or muscle fatigue (ii) Arthralgia and/or arthritis (iii) Chronic fatigue and sleep disturbance (iv) Neurological manifestations associated with demyelination (v) Cognitive decline and memory loss (vi) Pyrexia and dry mouth (vii) Improvement of symptoms after extraction of the inducing agent (viii) Positive biopsy of affected tissues	Other clinical manifestations (irritable colon syndrome) Presence of HLA (HLA DRB1 and HLA DQB1) Evolution of an autoimmune disease (multiple sclerosis and systemic sclerosis)

## Data Availability

The authors confirm that the data supporting the findings of this study are available within the article.
